# Screen Time and Autism: Current Situation and Risk Factors for Screen Time Among Pre-school Children With ASD

**DOI:** 10.3389/fpsyt.2021.675902

**Published:** 2021-08-06

**Authors:** Han-Yu Dong, Jun-Yan Feng, Bing Wang, Ling Shan, Fei-Yong Jia

**Affiliations:** Department of Developmental and Behavioral Pediatrics, The First Hospital of Jilin University, Changchun, China

**Keywords:** screen time, ASD, language development, developmental quotient, autistic symptoms, parent-child interaction, risk factors

## Abstract

**Objective:** To investigate the current status of screen time in children with ASD, its correlation with autistic symptoms and developmental quotient (DQ), and the factors affecting screen time.

**Method:** One hundred ninety-three Chinese children with ASD were recruited. We collected the demographic and screen time data using a questionnaire. The ASD core symptoms and developmental quotient (DQ) were measured by the Autism Behavior Checklist (ABC), Childhood Autism Rating Scale (CARS), Autism Diagnostic Observation Schedule-Second Edition (ADOS-2), Griffiths Development Scales-Chinese Language Edition (GDS-C), and Chinese Children's Parent-Child Relationship Questionnaire (CPCIS). Then, we analyzed the correlations between the screen time of children with ASD and the ABC, CARS, ADOS, GDS-C DQs, and CPCIS scores. Linear regression was used to analyze the risk factors that affect screen time.

**Results:** The children's average daily screen time was 2.64 ± 2.24 h. Forty eight percent children were exposed to two or more types of electronic devices. Their favorite activity of screen time was watching cartoons. Only 34% children spent screen time accompanied by parents and with communication. 50.26% children had no screen time before sleeping. The screen time of children with ASD had a negative correlation with the GDS-C CQ (*r* = −0.234, *P* = 0.001) and the CPCIS score (*r* = −0.180, *P* = 0.012) and a positive correlation with the CARS score (*r* = 0.192, *P* = 0.009). A low father's education level (*P* = 0.010), less restriction of the child's screen time by the guardian (*P* = 0.001), greater caregiver screen time (*P* < 0.001), the use of the screen as a tool for child rearing (*P* = 0.001), and the child's ownership of independent electronic equipment (*P* = 0.027) are risk factors for long screen time in children with ASD.

**Conclusion:** The screen time of children with ASD in China is higher than the recommended standard, and the current situation is serious. The screen time of ASD children is related to their autism symptoms, DQ and parent-child interaction. Low paternal education levels, less restriction of children's screen time by guardians, greater guardian screen time, the use of screens in child rearing, and children's ownership of independent electronic equipment can lead to an increase in children's screen time. These findings may have implications for family intervention strategies.

## Introduction

Autism spectrum disorder (ASD) is a neurodevelopmental disorder characterized by persistent deficits in social communication and interaction and stereotyped or repetitive patterns of behavior, interests or activities. In recent years, its prevalence has been increasing. The latest report has revealed a prevalence rate as high as 1 in 54 (1), making it a common disorder that seriously endangers children's social adaptability. Currently, it is generally believed that genetic and environmental factors and their interactions lead to the phenotypes of autism; however, the exact causal mechanism remains uncertain (2). The reported increase in prevalence in ASD is partly due to changes in diagnostic criteria, reductions in the misdiagnosis rate, and increases in the consultation rate, but the reasons for the increase are not limited to these factors. Environmental factors may play an important role in the pathogenesis of ASD (3) and may include socioeconomic factors, nutritional factors, heavy metal exposure, and air pollution. In ASD patients, a potential environmental factor is electronic screen time (4). Screen time has adverse effects on both children with ASD and children with typical development. These adverse effects include poor academic performance (5), sleep problems (6), attention problems (7), social behavior deficits (8), obesity (9), adverse cardiovascular events (10), language delay (11), mood disorder (12), and even autistic-like symptoms (13).

The American Academy of Pediatrics (AAP) has recommended that for children younger than 2 years old, exposure to digital devices should be avoided; moreover, screen time should be limited to 1 h per day for 2–5-year-olds (14). However, according to reports in the literature, the screen exposure time of many children, including children with typical development (15, 16) exceeds the AAP recommended standard. In the population of children with ASD, this phenomenon may be more serious. A systematic literature review conducted in 2019 (17) showed that children with ASD spent more time watching television than the typical control group. A Thai study on the screen time of young ASD children (average age 2.56 years) (18) showed that their average screen time was 4.60 h.

Studies have found that early exposure to electronic screens can cause neurochemical and anatomical changes in the brain, and neurotransmitter changes may cause behavioral problems in children (19). Research on the relationship between screen time and behavior in ASD children (13) shows that children who are exposed to electronic screens for more than 3 h a day have language delay, attention deficit and hyperactivity problems. Krupa's research has indicated that the screen time of ASD children and of their family members could affect the interaction between mother and child (20). These poor social interactions (mainly games) can further exacerbate children's autism-like symptoms and affect their development.

At present, studies on the factors affecting the screen time of children with ASD are not rare (15, 21), but the screen time of children is very closely related to region and social systems, and there is no relevant research from China. Therefore, research on the screen time of children with ASD is of great significance for understanding the current situation of children with ASD and identifying the risk factors for long screen time in these children. Such research could provide further evidence for limiting the screen time of children with ASD, thereby helping to provide clinical strategies for the families of children with ASD to improve ASD symptoms, language development and parent-child interaction.

Our team's preliminary small-sample cross-sectional study on this issue (22) showed that the screen time of children with ASD is significantly longer than that of children with typical development and is significantly correlated with children's performance on developmental tests (especially of language ability). This correlation is more pronounced in children with ASD who are younger and are exposed to longer screen times.

We conducted this study to further explore the current status of the screen time of children with ASD in China; verify the relationship between their screen time and their developmental quotient, parent-child interactions and autistic symptoms; and identify the risk factors that affect their screen time.

## Methods

### Participants

A total of 193 children with ASD (157 boys and 36 girls, 81.34 vs. 18.66%) diagnosed for the first time in the Department of Developmental and Behavioral Pediatrics of the First Hospital of Jilin University between April 2020 and September 2020 were recruited for this study. Their ages ranged from 17 to 76.5 months (37.39 ± 10.96 months). The DSM-5 and ADOS-2 (The Autism Diagnostic Observation Schedule–Second Edition) were utilized for the diagnosis of ASD without systemic intervention. Children with severe physical disability, cardiopulmonary disease or uncontrolled epilepsy and other serious physical conditions were excluded. The study protocol was approved by the ethics committee of our hospital, and informed consent was provided by the parents or caregivers of the children who participated.

### Procedure

We investigated the children's socioeconomic and demographic profile; their current screen time experience, using the self-made Children's Screen Time Questionnaire; and their parent–child interaction, using the CPCIS (Chinese Parent–Child Interaction Scale). The children's ASD symptoms and developmental level were also examined using the Autism Behavior Checklist (ABC), Childhood Autism Rating Scale (CARS), and Griffiths Development Scales-Chinese Language Edition (GDS-C).

Screen time data were calculated. The correlations between screen time and the scores on the ASD-related scales, CPCIS and GDS-C were analyzed. The children were divided into subgroups according to screen time and age, and correlation studies of each subgroup were performed separately. Finally, the risk factors affecting the screen time of children with ASD were identified.

The socioeconomic and demographic information that was collected included name, gender (male or female), age, birth date (year, month and day), place of residence (urban or rural area), caregivers (parents or others), siblings, parents' education levels, household income, maternal post-partum depression (yes or no) and outdoor activity time (hours).

The content of the self-made Children's Screen Time Questionnaire is as follows: screen time on weekdays and weekends, the type of electronic screen (TV, smart phone, computer, etc.), the main activity during screen time (cartoons, video games, Chinese poems, learning, etc.), parental accompaniment during screen time (accompaniment with communication, accompaniment without communication and no accompaniment), frequency of electronic screen use before going to bed, changes in screen time exposure in the past month (increased, decreased, or unchanged), restrictions on screen time (restricted, partially restricted, or not restricted), screen time of caregivers, age at first screen exposure, use of electronic screens as a tool to raise children (always, sometimes, or barely), use of screen time as a reward or punishment (always, sometimes, or barely), and independent ownership of electronic equipment (yes or no). The evaluator calculated and recorded the average daily screen time of the children using the following formula: average daily screen time (h) = [screen time per day on weekdays (h) × 5+screen time per day on weekends (h) × 2]/7.

The CPCIS was designed based on the literature and clinical observations in the Chinese context. It is an easily administered, valid, and reliable tool for the assessment of parent–child interactions in Chinese families (23). It includes eight items that have good validity and reliability. These items related to reading, drawing, singing, storytelling, discussing news and affairs, arithmetic and mathematics, English letters, and Chinese characters. Every item is divided into 0–5 points according to the frequency of interaction between children and parents per week (representing 0 to 5 times per week).

The ADOS-2 was utilized in this study as a diagnostic tool for ASD. The ADOS-2 is a semistructured, standardized assessment tool for individuals with suspected ASDs and measures autism symptoms in the domains of social relatedness, communication, play, and repetitive behaviors; it is deemed to be a gold standard for ASD diagnostic evaluation (24). The ADOS-2 modules 1 and 2 have calibrated severity scores: scores of 3 and 4 indicate low-level evidence, scores of 5–7 indicate moderate-level evidence and scores of 8–10 indicate high-level evidence. The ADOS-2 Toddler Module, which is used for children younger than 31 months, has no calibrated severity scores.

The symptom evaluation scales included the ABC and the CARS. The ABC is a 57-item screening checklist for autism containing 5 subscales (body behavior, sensory, self-care, language and social interaction). It is designed for parent interviews. The CARS was developed by Schopler and Reichler et al. and is used as a diagnostic scale. It consists of 15 subscales, each of which is scored on a continuum from normal to severely abnormal. The CARS requires observation of the behavior of ASD children in a consulting room. The reliability and validity of the ABC and CARS in the Chinese context are adequate, reflecting the scales' utility for the clinical diagnosis and evaluation of ASD symptoms.

The GDS-C is a popular tool in the Chinese social context and has good reliability and validity (25). It uses five independent subscales to assess the development level of children aged 0–2 years: physical mobility (A scale), personal social skills (B scale), hearing-speech (C scale), eye-hand coordination (D scale) and performance (E scale). Children aged 3–8 are also tested to assess their practical reasoning (F scale). The test scores are converted to a developmental age (DA) according to the Chinese norm for the GDS-C; the chronological age (CA) is calculated as the date of assessment minus the date of birth; and the development quotients (DQs) = DA × 100/CA (26). The DQs of each scale are called the AQ, BQ, CQ, DQ, EQ, and FQ.

### Statistical Methods

SPSS 23.0 statistical software was used for statistical analysis. Continuous variables with normal distributions are represented as the means ± standard deviations (SDs), and continuous variables with abnormal distributions are represented as the medians (P25–P75). Categorical variables are represented as frequencies (percentages).

The current screen time situation was analyzed using the Descriptive Statistics function in SPSS. Spearman's rank correlation was used for the two non-normally distributed variables in the correlation study. A linear regression model was used to analyze the risk factors for screen time. The results were considered significant at p < 0.05.

## Results

### Current Situation of Screen Time Among Children With ASD

**Figure 1:**
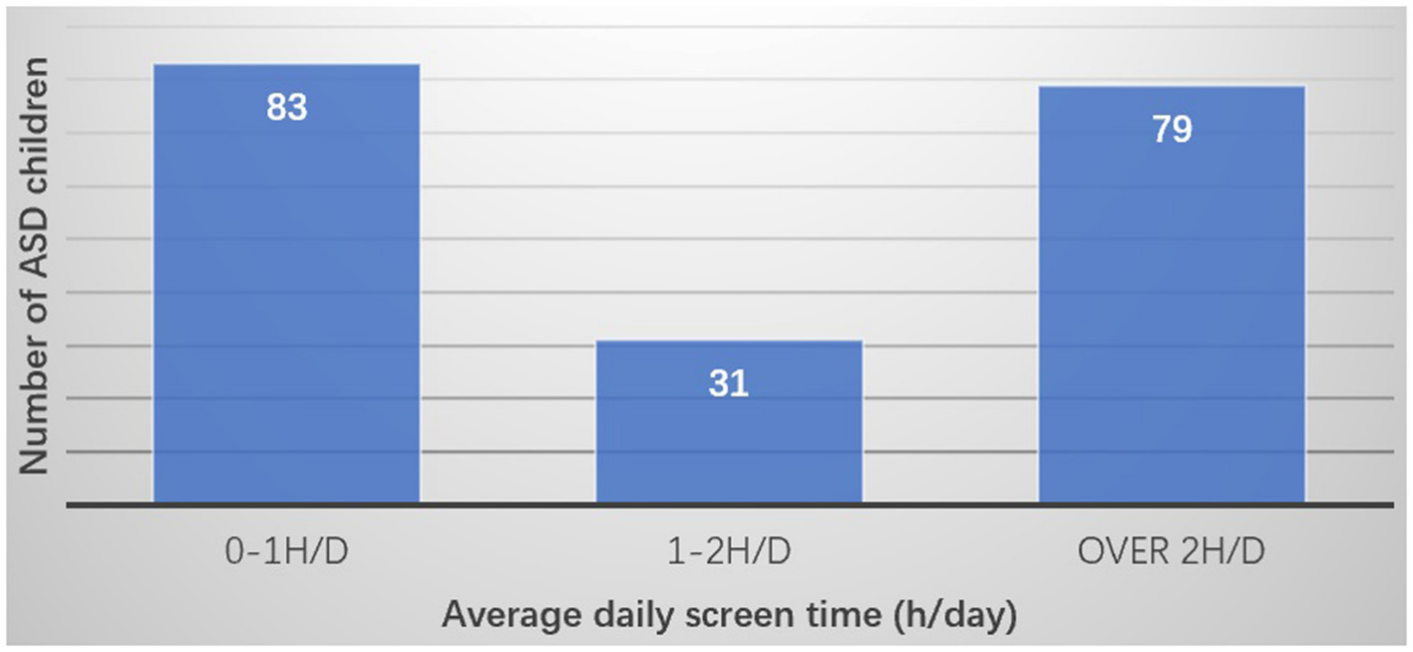
Average daily screen time.

The average screen time of children with ASD on weekdays was 2.68 ± 2.33 h, and that on weekends was 2.57 ± 2.29 h. Children with ASD seemed to have longer screen time on weekdays than weekends, but the difference was not significant (*P* = 0.674). The average daily screen time of children across the entire week was 2.64 ± 2.24 h: *n* = 83 (43.0%) children had 0–1 h, *n* = 31 (16.1%) children 1–2 h, and *n* = 79 (40.9%) children more than 2 h of screen time (Figure 1).

**Figure 2:**
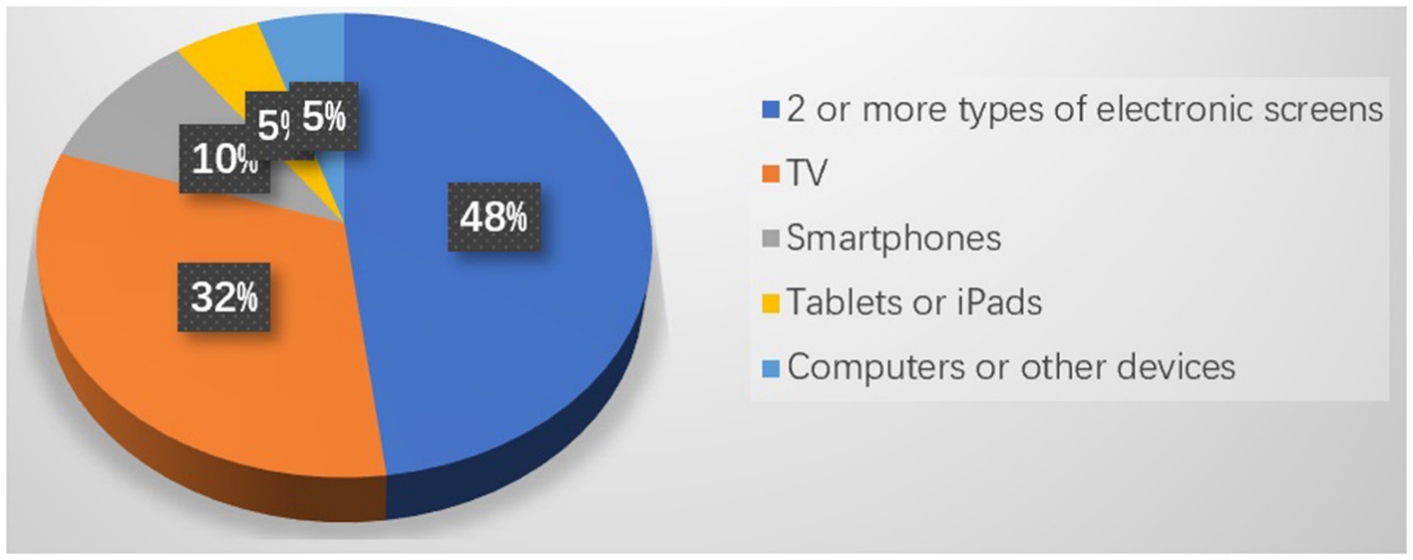
Distribution of electronic screen type in Chinese children with ASD.

The results for the type of electronic screen were as follows: *n* = 93 (48%) of children were exposed to 2 or more types of electronic screens daily. *N* = 61 (32%) of children's screen time was occupied by TV watching, *n* = 20 (10%) by smartphone use, and another 10% by the use of tablets or iPads, computers or other devices (see details in Figure 2).

**Figure 3:**
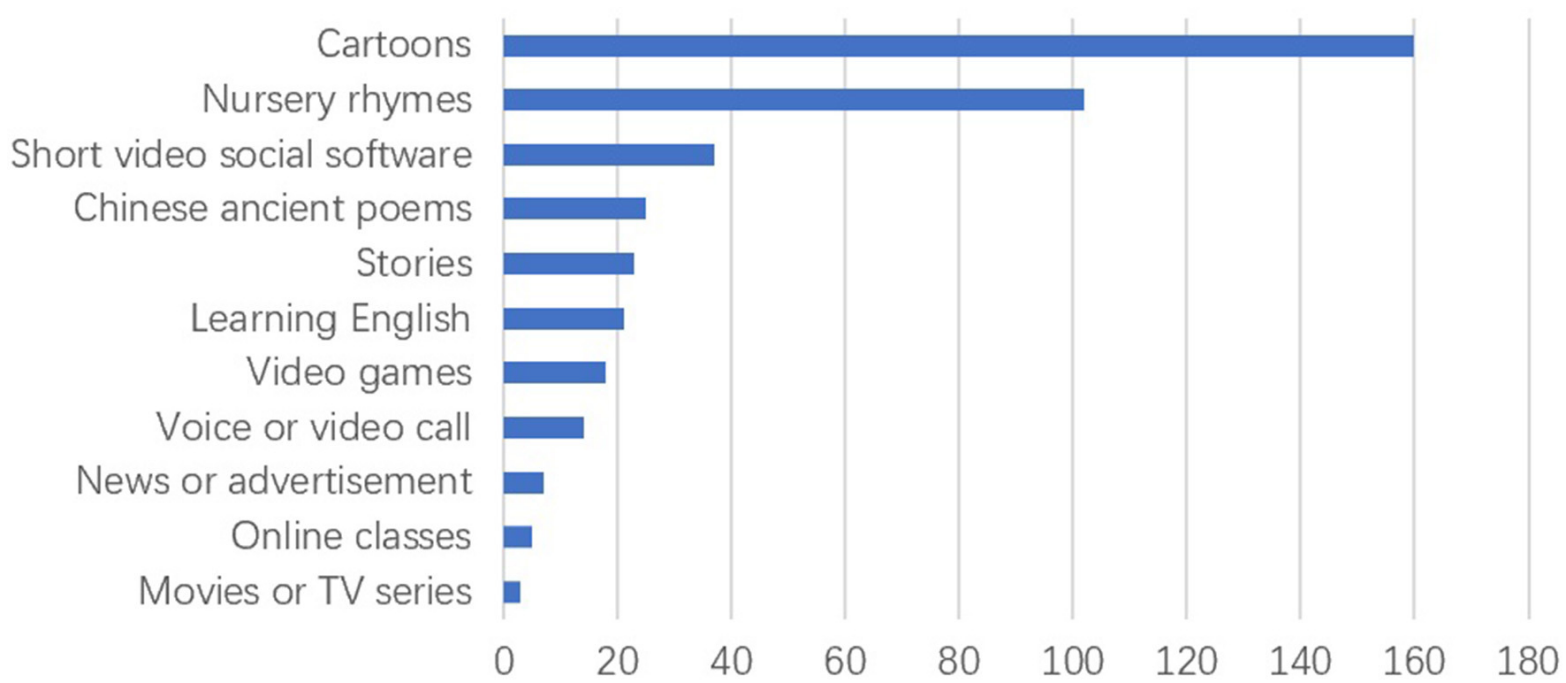
Main activity during screen time.

The results showed the following regarding the main activity during screen time: *n* = 160 (83%) children with ASD watched cartoons, *n* = 102 (53%) watched nursery rhymes, and *n* = 37 (19%) watched short videos on social platforms (TikTok, etc.). Other screen time activities included reading ancient Chinese poems (*n* = 25), reading stories (*n* = 23), learning English (*n* = 21), playing video games (*n* = 18), making voice or video calls (*n* = 14), reading news or advertisements (*n* = 7), taking online classes (*n* = 5), and watching movies or TV series (*n* = 3) (Figure 3).

We investigated parents' involvement in children's screen time: *n* = 65 (34%) children spent screen time accompanied by parents and with communication, *n* = 52 (28%) were accompanied by their parents but without communication and *n* = 72 (38%) were not accompanied by parents.

Regarding the frequency of the use of electronic screens before going to bed, *n* = 97 (50.26%) children had no screen time before sleeping, *n* = 41 (21.24%) had screen time before bed 1 to 3 times a week, *n* = 16 (8.29%) 3 to 5 times a week, and *n* = 39 (20.21%) more than 5 times a week. In the past month, *n* = 15 (7.78%) children's exposure to screen time increased, *n* = 77 (39.90%) children's screen time remained unchanged, and *n* = 101 (52.32%) children's screen time decreased.

### The Correlations of Screen Time With ASD Symptoms, Development Quotients and Parent–Child Interactions in Children With ASD

The results of the Spearman rank correlation test showed that the screen time of children with ASD had a negative correlation with the GDS-C CQ (*r* = −0.234, *P* = 0.001) and CPCIS score (*r* = −0.180, *P* = 0.012) and a positive correlation with the CARS score (*r* = 0.192, *P* = 0.009) (see Table 1).

**Table 1:** The correlations of screen time with ASD symptoms, development quotients and parent–child interaction.

	**ABC**	**CARS**	**ADOS**	**AQ**	**BQ**	**CQ**	**DQ**	**EQ**	**FQ**	**CPCIS**
*r*	0.021	0.192	−0.100	<0.001	−0.047	−0.234	0.041	0.047	−0.143	−0.180
*P*	0.779	0.009^*^	0.257	0.996	0.524	0.001^*^	0.577	0.522	0.559	0.012^*^

* *P < 0.05*.

We divided the children with ASD into a longer screen time subgroup and a shorter screen time subgroup based on the cutoff recommended by the AAP (27) (2.0 h/d). The longer screen time subgroup had 83 children with ASD, and the average screen time was 4.89 ± 1.61 h/d. The shorter screen time subgroup had 110 children with ASD, and the average screen time was 0.98 ± 0.65 h/d. We conducted the aforementioned correlation study. The results showed that among children with ASD with a screen time longer than 2.0 h per day, screen time had a positive correlation with the CARS score (*r* = 0.317, *P* = 0.004) and had a negative correlation with the CQ of the GDS-C (*r* = −0.220, *P* = 0.050) and the CPCIS score (*r* = −0.289, *P* = 0.008). However, we did not find any correlation between screen time and the ASD-related scale scores or the DQs in the shorter screen time subgroup (see details in Table 2).

**Table 2:** The correlations of screen time with the ASD-related scale scores, the development quotients and parent–child interaction in the longer and shorter screen time subgroups.

	**Longer ST subgroup (≥2 h/day)**		**Shorter ST subgroup (<2 h/day)**	
	***r***	***P***	***r***	***P***
ABC score	−0.020	0.863	0.162	0.092
CARS score	0.317	0.004^*^	0.159	0.109
ADOS severity score	0.235	0.074	0.201	0.090
AQ	0.088	0.437	0.104	0.291
BQ	−0.078	0.491	−0.088	0.370
CQ	−0.220	0.050^*^	−0.144	0.144
DQ	−0.017	0.879	−0.021	0.835
EQ	0.052	0.644	−0.052	0.600
FQ	−0.501	0.169	−0.273	0.445
CPCIS	−0.289	0.008^*^	−0.032	0.743

* *P < 0.05*.

In addition, we divided the ASD group into an older group and a younger group according to the average age (36.5 months). The screen time of the younger subgroup (*N* = 110, average age = 29.70 ± 3.90 m) was 2.69 ± 2.32 h, and that of the older subgroup was 2.59 ± 2.20 h (*N* = 83, average age = 47.59 ± 8.73 m). There was no significant difference in screen time between the two subgroups (*t* = 0.331, *P* = 0.741). The results of the correlation study showed that in the younger subgroup, screen time had a positive correlation with the CARS score (*r* = 0.488, *P* < 0.001). In addition, screen time in the younger subgroup had a negative correlation with the CQ of the GDS-C (*r* = −0.330, *P* = 0.001) and the CPCIS score (*r* = −0.217, *P* = 0.024). However, we did not find any correlation between screen time and the ASD-related scale scores or the DQs in the older subgroup (see details in Table 3).

**Table 3:** The correlations of screen time with the ASD-related scale scores, the development quotients and parent–child interaction in the younger and older subgroups.

	**Younger subgroup (<36.5 m)**		**Older subgroup (≥36.5 m)**	
	**r**	***P***	**r**	***P***
ABC score	0.165	0.092	−0.147	0.188
CARS score	0.488	<0.001^*^	−0.109	0.329
ADOS severity scores	/	/	−0.079	0.486
AQ	−0.124	0.203	0.170	0.138
BQ	−0.103	0.292	0.024	0.833
CQ	−0.330	0.001^*^	−0.099	0.390
DQ	0.005	0.959	0.088	0.443
EQ	0.041	0.674	0.055	0.632
FQ	/	/	−0.026	0.923
CPCIS	−0.217	0.024^*^	−0.130	0.242

*Children under 31 months of age use the M-T version of the ADOS, and there is no severity score. Therefore, in the younger subgroup, more than half of the children had no severity score data, so no statistical analysis was performed. In the GDS-C, FQ scores are not available until the F field ability exceeds 24 months. Therefore, most children in the younger subgroup had no scores in the F field of the GDS-C; therefore, statistical analysis was not performed*.

* *P < 0.05*.

### Risk Factors of Screen Time in Children With Autism

#### Univariate Analysis Results of Factors Related to Screen Time

The Spearman rank correlation results for continuous variables showed that the screen time of children with ASD was negatively correlated with the CQ of the GDS-C and the CPCIS score (Table 1), and there was no correlation between screen time and age (*r* = 0.025, *P* = 0.730).

For categorical variables, the Mann-Whitney U test is used for two independent samples, and the K independent samples K-W non-parametric test is used for multiple independent samples. The results showed that degree of education of the mother (χ2 = −2.113, *P* = 0.035) and father (χ2 = −3.139, *P* = 0.002), outdoor activity time (χ2 = −2.283, *P* = 0.022), whether screen time was restricted (*Z* = 33.328, *P* < 0.001), caregiver screen time (*Z* = 40.771, *P* < 0.001), the use of electronic screens as a tool to raise children (*Z* = 51.285, *P* < 0.001), the use of screen time as a reward or punishment (*Z* = 11.357, *P* = 0.003) and ownership of independent electronic equipment (χ2 = −3.261, *P* = 0.001) were closely related to the screen time of children with ASD (see details in Table 4).

**Table 4:** Univariate analysis results of the factors related to screen time for the categorical variables.

	***N***	**Screen time (M ± SD) (h/day)**	**Median (interquartile range)**	**Z/χ2**	***P***
**Gender**				−0.510	0.610
Male	157	2.63 ± 0.18	2.00 (3.43)		
Female	36	2.72 ± 0.36	2.00 (3.00)		
**Place of residence**				0.085	0.958
Urban	141	2.69 ± 0.19	2.00 (3.36)		
Township	25	2.55 ± 0.43	2.00 (3.38)		
Rural	27	2.66 ± 0.50	2.00 (3.50)		
**Caregivers**				−0.558	0.577
Parents	132	2.57 ± 0.19	2.00 (3.36)		
Others	61	2.90 ± 0.35	2.00 (3.36)		
**Maternal education**				−2.113	0.035^*^
Senior high school or below	106	2.96 ± 0.22	2.29 (3.00)		
Junior college or above	87	2.26 ± 0.23	1.50 (3.50)		
**Paternal education**				−3.139	0.002^*^
Senior high school or below	112	3.02 ± 0.21	3.00 (3.00)		
Junior college or above	81	2.21 ± 0.25	1.00 (2.86)		
**Annual income (10,000 yuan)**				2.631	0.452
0–5	48	2.82 ± 0.31	2.85 (3.13)		
5–10	96	2.58 ± 0.24	2.00 (3.50)		
10–20	33	2.97 ± 0.44	2.00 (3.00)		
Above	16	1.94 ± 0.48	1.00 (2.67)		
**Post-partum depression**				−0.115	0.909
Yes	31	2.47 ± 0.37	2.00 (3.36)		
No	162	2.72 ± 0.18	2.00 (3.36)		
**Siblings**				−0.652	0.514
No	132	2.59 ± 0.19	2.00 (3.36)		
Yes	61	2.79 ± 0.29	2.00 (3.00)		
**Outdoor activities**				−2.283	0.022^*^
<1 h	84	3.07 ± 0.26	2.57 (3.43)		
More than 1 h	109	2.31 ± 0.21	1.71 (3.50)		
**Screen time restriction**				33.328	<0.001^*^
No restriction	29	4.52 ± 0.53	4.00 (4.00)		
Partial restriction	100	2.88 ± 0.21	2.00 (3.00)		
Restriction	64	1.53 ± 0.19	1.00 (1.50)		
**Screen time of caregivers**				40.771	<0.001^*^
0–1 h	58	1.47 ± 0.21	0.64 (1.79)		
1–2 h	38	1.91 ± 0.29	1.29 (1.89)		
2–4 h	52	3.27 ± 0.32	3.28 (2.07)		
>4 h	44	4.01 ± 0.38	4.00 (4.00)		
**Age at first screen exposure (years)**				7.251	0.064
<1	79	2.48 ± 0.27	1.71 (3.50)		
1–1.5	67	3.09 ± 0.25	3.43 (3.00)		
1.5–2	24	1.97 ± 0.40	1.00 (5.83)		
>2	23	2.67 ± 0.59	2.00 (3.89)		
**As a tool to raise children**				51.285	<0.001^*^
Almost never	60	1.49 ± 0.24	0.64 (1.43)		
Sometimes	91	2.53 ± 0.18	2.00 (3.00)		
Always	42	4.76 ± 0.39	4.43 (3.52)		
**As a reward or punishment**				11.357	0.003^*^
Almost never	109	2.33 ± 0.21	1.29 (3.50)		
Sometimes	58	2.66 ± 0.27	2.00 (3.00)		
Always	26	4.05 ± 0.56	4.00 (4.00)		
**Ownership of independent electronic equipment**				−3.261	0.001^*^
No	156	2.40 ± 0.17	2.00 (3.50)		
Yes	37	3.80 ± 0.41	4.00 (3.43)		

* *P < 0.05*.

#### Linear Regression Results for the Risk Factors for Screen Time in Children With ASD

The statistically significant factors in the univariate analysis were incorporated into the linear regression model. The continuous variables were the CQ of the GDS-C and the CPCIS score. The categorical variables were coded as follows: Maternal and paternal education (senior high school or below = 1, junior college or above = 2), outdoor activities (<1 h = 1, ≥1 h = 2), screen time restrictions (no restrictions = 1, partial restriction = 2, restriction = 3), screen time of caregivers (0–1 h = 1, 1–2 h = 2, 2–4 h = 3, >4 h = 4), use as a tool to raise children (almost never = 1, sometimes = 2, always = 3), use as a reward or punishment (almost never = 1, sometimes = 2, always = 3), ownership of independent electronic equipment (no = 1, yes = 2). A low paternal education level and no restriction of children's screen time are risk factors for long screen time in children with ASD. Low caregiver screen time, not using electronic equipment as a tool to raise children and not owning independent electronic equipment are protective factors for long screen time in children with ASD (see details in Table 5).

**Table 5:** Linear regression results of the risk factors for screen time in children with autism.

	***B***	***t***	***P***
Paternal education	−0.705	−2.607	0.010^*^
Screen time restriction	−0.787	−3.413	0.001^*^
Screen time of caregivers	0.626	5.054	<0.001^*^
As a tool to raise children	0.757	3.243	0.001^*^
Ownership of independent electronic equipment	0.792	2.238	0.027^*^

* *P < 0.05*.

## Discussion

### Current Situation of Screen Time Among Children With ASD

#### Screen Time

Our results suggest that the current situation of autistic children with a long screen time is serious. A total of 40.9% of children with ASD have an average daily screen time of more than 2 h a day. This result is consistent with previous studies. Mazurek and Wenstrup (28) investigated the screen time of children with ASD (8–18 years old) and found that the average screen time was 2.4 h per day for boys and 1.8 h per day for girls. Healy et al. (29) also found that 13-year-old children with ASD spend an average of 121–150 min a day watching TV. MacMullin (30) found that individuals with ASD were reported to spend an average of 5.67 h per day using electronic devices from the ages of 6 to 21 years old. However, there are relatively few studies on younger ASD groups. According to a study in Arabic-speaking countries in the Middle East (31), the average screen time of children with ASD before the age of 3 was 4.7 h a day in Qatar. This is longer than the average screen time of 2.64 h per day for ASD children in China. Our results also show that the screen time on weekdays has a tendency to be longer than that on weekends (although the different is not statistically significant), which may be related to the increase in parent-child interaction when parents are at home on weekends. However, Must's research (32) suggests the opposite, reporting that children with ASD's screen time on weekends is longer than that on weekdays. The reason for this difference may be that Must's sample covered a larger age range (3–11 years vs. 17 months to 76.5 months) and had a higher average age (6.6 years vs. 37.39 months) than the sample in this study. However, older children often receive special education or schooling on weekdays, and weekends are used as rest time, giving them more opportunity to for electronic screen time. Therefore, we will continue to follow up with children with ASD to determine the status of electronic screen use in these children at school age in China.

#### Types of Electronic Devices

Our research found that 48% of children with ASD are exposed to more than two types of electronic screens. This is also consistent with existing reports. MacMullin (30) reported that children with ASD are exposed to more than three electronic devices on average. Children with ASD engage in a variety of screen activities, which reflects their preference for such activities. In 2014, Kuo (33) identified TVs and computers as the favorite electronic devices of teenagers with ASD, while the preferred electronic devices of children with ASD in our study were TVs and smartphones. Almost all studies report that TV is the most-used electronic device among children. This may be due to the wide availability of and larger audience for TV. With the development of network technology and advancements in electronic technology, smartphones play an important role in the screen time of children with ASD.

#### Main Screen Activities

Games and social media were the main screen activities of children with ASD in MacMullin's study (30) while the main screen activities of children with ASD in our study were cartoons (*n* = 160, 83%), nursery rhymes (*n* = 102, 53%) and short videos on social platforms (*n* = 37, 19%). This may be related to the younger age of the children in this study. Older adolescents with ASD have certain learning, language and social abilities, all of which change with age. There are no reports on the screen time of Chinese teenagers and young adults with ASD. This issue needs further follow-up and investigation in the Chinese context.

We also found that some screen activities were related to children's learning behaviors and involved reading ancient Chinese poems (*n* = 25, 13%), learning English (*n* = 21, 11%) and taking online classes (*n* = 5, 2.6%). Therefore, we must also pay attention to whether there is a potential positive effect of screen activity. Shane (34) reported that the use of electronic screens can provide a more attractive learning environment for children with ASD. Electronic screen media may be an effective tool for teaching or improving the social, language, and cognitive skills of children with ASD. However, we have reservations about this possible positive effect. As children develop, they continuously gain a variety of new knowledge and apply it to different situations, which is called the transfer of learning (35). Studies have shown that children under the age of 3 experience difficulty transferring information from 2D contexts to 3D real contexts. Even if the 2D learning context includes touchscreen puzzle interaction, which is better than video games, it may still cause interference with learning, and the transfer deficit may still exist (36). This may be due to children's immature perceptual encoding and inability to comprehend symbolic representation (36). Therefore, young children learn less from television, touchscreen computers and books than they do from face-to-face communication and contact. Early childhood learning, including language learning, depends to a large extent on the direct influence of the language context in social interaction rather than on the input of electronic products (13).

#### Parents' Attitudes

Our research found that only *n* = 65 (34%) of parents are present with their children and communicate with them during the children's screen time. This statistic is consistent with Hermawati's report (13), which found that more than half of children (66.6%) were exposed to electronic screens without parent-child interaction. The results of a large sample study published in China on pre-school children with normal development in Shanghai showed that only 26% of parents were able to accompany their children during screen time and talk about the content. Although many parents are aware of the hazards of screen exposure, quite a few parents tend to use electronic screens as temporary “electronic babysitters” so that they can complete housework, leaving children to spend their screen time alone. Parents of children with ASD report that television and videos are often used to distract, prevent tantrums, and control behaviors. In discussions of children with disruptive behavioral disorders (DBDs), parents tended to regard screen time as an opportunity for “peace and quiet” (21). Our research also found that only *n* = 40 (21%) parents did not use electronic screens as a tool for raising children, and only *n* = 64 (33%) parents strictly limited children's screen time. Therefore, in response to this problem, the AAP recommendations should be followed; these advocate watching high-quality programs and suggest that parents watch TV with their children and help them understand the content on the screen and apply it in the real world (14).

#### Sleep

In our research, electronic screens were frequently used before going to bed, and ~50% of children had screen time before sleeping. Currently, there are reports suggesting that screen time before going to bed is associated with sleep reduction in children with autism (37) and affects sleep quality (6, 38). The possible reasons for the mechanism by which screen time affects sleep are as follows: delayed sleep time (39), psychological, emotional, and/or physiological arousal (40), inhibition of the level of melatonin by screen illumination (41), and sleep rhythm disruption. Sleep has an impact on mood, behavior, learning and memory (38). Therefore, controlling screen time may be helpful in children with ASD who have sleep problems. This study investigated only the use of electronic screens before going to bed, and the impact on sleep needs further research and discussion.

### Screen Time Is Related to ASD Symptoms, the Development Quotient and Parent–Child Interaction

Our study showed that screen time is related to autistic symptoms, the hearing-speech development quotient (CQ) and parent–child interaction in children with ASD. The correlation may be more pronounced in children with ASD who have longer screen time and in younger children with ASD. This result is consistent with our previous small-sample research results and other existing studies.

#### Screen Time Is Related to Autistic Symptoms

Adverse environmental factors have an important effect on the clinical symptoms of children with ASD. Excessive screen time has adverse effects on children's social participation and behavior regulation (30). Yousef's study found that screen time > 2 h/d among school-age children was positively correlated with children's autism-like symptoms (42). Subgroup analysis showed that the correlation between screen time and symptoms is more obvious in the younger subgroup and the subgroup with longer screen time, which is in line with our expectations. ASD is closely related to brain development. Studies have shown that in the early post-natal period or even before birth, the brains of children with ASD have multiple neurological defects. The brain develops rapidly in the early stages after birth and is influenced by the developmental environment (43). Therefore, we speculate that the longer that children with autism are exposed to unfavorable environmental factors and the less mature their brains are, the more obvious their developmental delay and autistic symptoms will be. From what has been discussed above, we conclude that long-term screen time can affect symptoms of autism as an adverse environmental factor.

#### Screen Time Is Related to the Hearing-Speech Development Quotient

Screen time was proven to be related to the development of language in children with ASD in this study. The younger the age and the longer the screen exposure time, the more serious the impact on language development is. Hermawati reported that exposure to electronic media in early life (<2 years old) had a negative impact on language (13). Chonchaiya et al. found that children who started watching TV as early as 12 months and who watch for more than 2 h a day are six times more likely to have language delays. The latest meta-analysis published by Madigan in 2020 (44) shows that screen use is related to children's language skills. The findings support pediatric recommendations to limit children's duration of screen exposure, to select high-quality programming, and to co-view when possible. These findings are all in line with our research results. It is important to provide family health guidance to parents of children with ASD. We remind parents of the importance of children's media ecology for children's language development, especially that of children with social communication problems. Recommendations regarding the frequency and duration of screen time may be conducive to optimizing children's development. Importantly, society and the relevant professionals have a responsibility to continue to promote and advocate children's screen time initiatives and supervise media platforms.

#### Screen Time Is Related to Parent-Child Interaction

We also found a negative correlation between the screen time of ASD children and the parent-child relationship. The longer the screen time is, the worse the parent-child interaction level. Krupa's study (20) also reported the same results. In addition to children's personal screen time, family screen time also has a negative impact on the parent-child relationship. Therefore, we should limit not only the screen time of children with ASD but also the screen time of their families (20). Some studies indicate that parent-child interaction is an intermediary factor. Prolonged screen time in children with ASD reduces the level of parent-child interaction, which affects the development of children's communication and social skills. If this factor is changed, the harmful effects of excessive screen time may be partially offset by improved parent-child interaction (45). This finding can be used to inform guidance for parents.

### Risk Factors for Screen Time in Children With ASD

Our results suggest that low paternal education levels, no restrictions on screen time, longer caregiver screen time, the use of media devices as a tool to raise children, and independent ownership of electronic equipment are risk factors for screen time in children with ASD. This result suggests that family factors are the main factors affecting the screen time of children with ASD. A survey (46) of primary and middle school students in 12 provinces and cities in China showed that sex, urban or rural residence, living in the west or east province of China, self-rated academic performance, parents' education levels and physical activity could affect children's screen time. This is slightly different from the results of our research. The daily life of typically developing primary and middle school students is greatly influenced by the school environment, but the children in our study were young and had ASD. Many children with ASD cannot receive education in a normal school setting, and the family environment has a greater impact than other environmental factors; this may explain the differences between the above-mentioned survey and our study.

A study of 18-month-old children in Sydney (15) showed that screen time >2 h is related to the following factors: mother has no partner, fewer than three siblings, father has a job, no outdoor equipment, and outdoor activity <5 times a week. Similar to the Sydney study, some studies (47) found that the presence of siblings may have an impact on children's screen time, but our research did not find this result. The reason may be that it was affected by the “family planning” policy implemented several years ago. Although China has gradually opened the two-child policy in 2015, there are still few Chinese children with siblings. Only 61of the 193 children with ASD in this study had siblings.

Healy's study (48) reported a relationship between lifestyle and screen time in children with ASD and found that screen time was related to less physical activity. Must's study (49) also emphasized that there is a negative correlation between physical activity time and the screen time of children with ASD. Our study did not investigate the physical activity time of children with ASD. This is a limitation of our research. We speculate that physical activity may be similar to parent-child interaction, which is also an intermediary factor of screen time effect on the development of children with ASD. A longer period of physical activity may offset the impact of screen time on children. This speculation needs to be further explored in follow-up research.

Healy (48) also found that screen time is correlated with parents' attitude toward not restricting screen time and toward the presence of a TV in their child's room. Similar results were found in our research: not restricting screen time was a risk factor for screen time in children with ASD. However, the children in our study were younger and often did not have their own rooms. The findings of this study indicate that children with independent electronic devices, such as mobile phones and tablets, are at risk of longer screen time.

Previous studies (21, 47, 50) have shown that screen time is negatively correlated with family income and parental education levels. Our research also indicated that as family income increased, the screen time of children with ASD tended to decrease. Although this trend was not statistically significant, there may be some hints for clinical work. Parents with better economic conditions tend to have a higher social class and may more consciously engage in parent-child interaction with their ASD children as a form of continuous family intervention rather than using electronic screens as a nanny. In addition, this study also identified the father's education level as a factor influencing the screen time of children with ASD, consistent with the results of previous studies.

According to our research, we will provide relevant psychological education to the parents of ASD children, and help them to make family intervention plans, which may have greater significance for the management of ASD children.

### Limitation

The age range of this study is small. Screen activities of children of different ages will change with their cognitive ability and school activities, and the influencing factors may also be different, which needs further discussion.

The relationship between sleep and electronic devices has not been discussed in detail, and this may be the direction of future research.

Studies have shown that outdoor activity time may be related to children's screen time. Our study did not investigate the physical activity time of children with ASD.

## Conclusion

The screen time of children with ASD in China is above the recommended standard, and the current screen exposure situation is serious. The screen time of children with ASD is related to their autism symptoms, DQ and parent-child interaction. This correlation is more pronounced in younger children with ASD and those with longer screen time. A low father's education level, less restriction of the child's screen time by the guardian, greater caregiver screen time, the application of the screen as a tool for child rearing, and the child's ownership of independent electronic equipment can lead to an increase in a child's screen time.

Identifying the risk factors for longer screen time in children with ASD can provide a basis for limiting the screen time of children with ASD, which is beneficial for the improvement of ASD symptoms, language development and parent-child interaction in ASD children. It is of great significance to clinical decision making.

## Data Availability Statement

The raw data supporting the conclusions of this article will be made available by the authors, without undue reservation.

## Ethics Statement

The studies involving human participants were reviewed and approved by the ethics committee of the First Hospital of Jilin University. Written informed consent to participate in this study was provided by the participants' legal guardian/next of kin.

## Author Contributions

H-YD: methodology, investigation, and writing—drafting the initial manuscript. J-YF: methodology, investigation, formal analysis, and some writing. BW: data curation and formal analysis and editing the manuscript. LS: investigation, formal analysis, and editing the manuscript. F-YJ: conceptualization, funding acquisition, supervision, and oversight and resources. All authors contributed to the article and approved the submitted version.

## Conflict of Interest

The authors declare that the research was conducted in the absence of any commercial or financial relationships that could be construed as a potential conflict of interest.

## Publisher's Note

All claims expressed in this article are solely those of the authors and do not necessarily represent those of their affiliated organizations, or those of the publisher, the editors and the reviewers. Any product that may be evaluated in this article, or claim that may be made by its manufacturer, is not guaranteed or endorsed by the publisher.
